# Prevalence of Cognitive Distortion Markers in a Suicide Prevention Chat Service: Mixed Methods Study

**DOI:** 10.2196/81213

**Published:** 2026-04-28

**Authors:** Marijn ten Thij, Saskia Mérelle, Renske Gilissen, Johan Bollen

**Affiliations:** 1 Department of Intelligent Systems, Tilburg School of Humanities and Digital Sciences, Tilburg University, Warandelaan 2, Tilburg, 5037 AB, The Netherlands; 2 Centre for Social and Biomedical Complexity, Indiana University Bloomington, Bloomington, IN, United States; 3 113 Suicide Prevention, Amsterdam, The Netherlands; 4 Instituut Psychologie, Faculteit der Sociale Wetenschappen, Leiden University, Leiden, The Netherlands; 5 Instituut voor Informatica, Faculteit Natuurwetenschappen, Wiskunde, en Informatica, University of Amsterdam, Amsterdam, The Netherlands

**Keywords:** cognitive distortions, online suicide prevention helpline, natural language processing, cognitive behavioral therapy, suicide prevention

## Abstract

**Background:**

Suicide helplines increasingly employ chat services to aid those in urgent need, but the wording and structure of text-driven exchanges may affect their effectiveness.

**Objective:**

Given the association of cognitive distortions with depression and anxiety, this study investigated their prevalence in the language of individuals seeking help from the Dutch 113 suicide helpline.

**Methods:**

We observed the prevalence of cognitive distortions for both help seekers and counselors in a large volume of chat sessions (N=71,148) of the Dutch 113 suicide chat helpline using natural language processing. The results were compared to 2 large collections of online text data from Dutch social media and web content.

**Results:**

We found that nearly all types of cognitive distortions are more prevalent in the language of help seekers compared to the control group of helpline counselors. Distortions of the personalizing, emotional reasoning, and mental filtering types were, respectively, 20.22, 7.87, and 4.53 times more prevalent among help seekers, revealing a distinct pattern of thought and language among individuals affected by suicidality.

**Conclusions:**

Our results raise the prospect of improving the effectiveness of online therapeutic interventions that target cognitive distortions through lexical analysis that detects the cognitive and lexical markers of suicidality.

## Introduction

### The Societal Burden of Suicide

Suicide exacts a staggering societal cost. Recent World Health Organization (WHO) estimates indicate that more than 700,000 individuals die of suicide each year and its worldwide mortality exceeds that of malaria, HIV/AIDS, war, and homicide [1]. Suicide is the 10th leading cause of death in the United States, where its economic costs are estimated to be over US $70 billion per year in lifetime medical expenses and loss of productivity alone, with similar numbers across the globe [2-7]. Moreover, suicide is a leading cause of death among young people in the Netherlands. Increasing suicide rates among young people have been observed [8].

Beyond deaths from suicide, many more individuals experience suicidal thoughts and intent. US Centers for Disease Control and Prevention (CDC) statistics indicate that, in 2018, a total of 10.7 million, 3.3 million, and 1.4 million American adults either seriously thought about suicide, made a plan, and/or attempted suicide, respectively [9]. The burden on minors and young adults is equally high. In 2019, a total of 18.8% of high school students reported seriously considering suicide and 8.9% reported an attempt [10].

### Analyzing Language From Chat-Based Suicide Prevention Helplines

Given these indicators of a global mental health crisis [11], the death toll of suicide, and the large population contemplating or attempting suicide, scalable and effective mitigation is of the utmost importance [12-14]. Here, we focus on suicide prevention helplines, which aim to provide immediate assistance and access to important resources [15] to those experiencing an imminent risk of suicide. Many telephone helplines have recently been augmented with online chat services to extend access, in particular for the young [16] who may prefer chat services over telephone conversations. Given their use of ubiquitous online chat applications, chat helplines can offer time-sensitive, real-time, cost-effective, and anonymous assistance to individuals reaching out at a time of urgent need [17]. They are for these reasons preferred over other channels, such as email, text messaging, voice calls, and face-to-face counseling [18]. A large-scale study of a national suicide prevention helpline showed that the chat functionality had been helpful for almost 70% of the users and that 45% felt less suicidal after the interaction(s) [19]. In addition, short-term improvements were recently observed in suicidal ideation and psychological risk factors after a chat conversation in another national helpline [20].

Thus far, research has focused on studying suicidal ideation from language, for example, through linguistic features from social media content [21-24], poetry [25], forums [23,24,26-28], or suicide notes [29-31] using tools, such as Linguistic Inquiry and Word Count [32]. Examples of such linguistic features that are extracted from these texts are self-to-relational word counts [33], linguistic accommodation [26], hopelessness [28], social disengagement [28], or categories of stress [31].

By definition, chat-based suicide helplines rely on text-driven communication, which, although it takes place in real time, does not include verbal cues, body language, intonation, and other important aspects of in-person communication. They require that respondents interpret the written language of help seekers, detect cues and indications of the help seeker’s state, and respond accordingly. However, it is not clear what these written language cues or features may be in the context of chat-driven suicide helplines; this remains an important knowledge gap. Recent efforts that have started to address this gap focus on providing suggestions for counselors based on previous chat sessions [34] or training counselors in motivational interviewing [35].

### Detecting Cognitive Distortions in Suicide Prevention Helplines

There exists an extensive literature on language features associated with depression and other internalizing disorders, which are known to be strongly comorbid with suicidal ideation and depression [36-38]. For example, Eichstaedt et al [39] showed that language features indicative of emotional distress, interpersonal dissatisfaction, and rumination can be leveraged by machine-learning algorithms to predict depression in Facebook users at a reasonable level of accuracy. Rude et al [40] showed that the language of individuals with depression is indeed marked by the prevalence of specific identifiable terms. However, the language of individuals with depression is not only marked by the presence of discrete features that may be useful in diagnostic classification tasks, but also by a style of thinking that is deeply implicated with the development of internalizing disorders such as depression.

According to the theory underlying cognitive behavioral theory, the cognitive triad and the cognitive mediation model [41-43], individuals with depression exhibit self-reinforcing thought patterns in which unrealistic and overly negative views about the world, the future, and oneself cause a cascade of detrimental cognitive, affective, and behavioral changes associated with internalizing disorders. These thought patterns are referred to as cognitive distortions and frequently described in terms of 12 general types, eg, “I will never amount to anything,” which in this case amounts to “fortune-telling,” “dichotomization,” and “personalizing.” Regardless of type, all cognitive distortions share the characteristic that they amount to unrealistic, exaggerated, and overly rigid thinking patterns.

Cognitive behavioral therapy (CBT), now the leading treatment of depression, anxiety, and suicide [44-49], considers cognitive distortions an important intervention target. In fact, the very effectiveness of CBT demonstrates the importance of the identification and mitigation of cognitive distortions in an individual seeking help with depression or anxiety. Previous research [50,51] demonstrated that the online language of individuals with depression is indeed marked by a higher prevalence of cognitive distortions than a random control sample. Since depression is strongly comorbid with suicide [36-38], here we investigate whether the written language of individuals seeking help with suicidal thoughts in a chat-based helpline is similarly impacted by the presence of cognitive distortions. If so, as indicated by the effectiveness of CBT [48,49], addressing cognitive distortions may be an important driver and intervention target for suicidality in this context.

We analyzed 6,349,202 chat messages recorded from July 2017 to June 2021, which were split between those written by either help seekers or the professional respondents of the 113 suicide helpline in the Netherlands. We found that markers of some types of cognitive distortions are 20-fold more prevalent in the language of help seekers than the 113 counselors. In fact, a comparison with the online language of individuals with depression and a random sample recorded on Twitter revealed a similar difference and indicates that Twitter language may be particularly enriched with cognitive distortions even in the absence of any diagnosed mental health issues.

## Methods

### Ethical Considerations

This study was approved by the Indiana University Bloomington Institutional Review Board under protocol 2007591998 “Retrospective language analysis of suicide prevention online chat content (Dutch 113 service),” which was reviewed specifically for this study and its research team. Help seekers did not receive any compensation for the inclusion of their sessions in our analysis. By participating in the chat sessions at 113, help seekers approve that these sessions can be used for research purposes, as stated in the terms and conditions.

As our analysis pertains to individual health-related information, we took additional steps to ensure the privacy of all individuals involved in the chat sessions. We deidentified all data by assigning each chat session a unique but anonymous label, for example, cl123456, to remove all identifying information. All times are reported in terms of time elapsed from the start of the conversation. All raw data are stored in a protected environment at 113 suicide prevention. Finally, all analyses were performed and reported in the aggregate, that is, without reference to the individuals that constitute the specific cohorts of the study.

### Data and Sample

We performed our analysis on chat sessions recorded by the secure chat platform of the 113 suicide prevention helpline in the Netherlands. On this platform, help seekers can reach out to 113 employees to discuss any topics related to suicide. For our analysis, we selected all chat sessions that passed the initial triage stage determining whether the help seeker was in a safe location before being passed to a professional counselor (a psychologist or a volunteer). Chat sessions have 3 types of participants: help seekers, 113 counselors, and the chat service system itself. The chat service system posts automated messages to indicate that it has taken a specific action; we exclude these messages from our analysis. Applying this criterion resulted in a large-scale dataset consisting of 71,148 chat sessions that were recorded between July 1, 2017, and June 1, 2021. In total, these chat sessions contain 3,316,873 messages posted by help seekers and 3,032,329 messages posted by 113 counselors. The average monthly number of forwarded chat sessions that we have included in our analysis is 1513 (95% CI 1199-1874), showing high levels of engagement with the 113 helpline.

This study investigated the differences between the written language of a cohort of 113 help seekers who are seeking urgent help with suicidal ideation or intent and the written language of a control cohort with little or no suicidality in the context of an online chat environment. Given the nature of the 113 helpline, we did not expect to find many help seekers with low levels of suicidality that could serve as a control cohort. In fact, an analysis of the responses to the questionnaire [35,52] that help seekers are required to fill in showed insufficient discrimination between low and high levels of suicidality to construct a control cohort (see section “Types of Help seekers as control” in Multimedia Appendix 1). Here, we chose to use the 113 counselors as a control group since they are expected to be least affected by suicidality and depression, while conversing with help seekers on the same platform about the same topic. For the remainder of the analysis, *h* denotes the help seeker cohort and *O* denotes the Counselor cohort.

In addition, we compared the written content of the 113 chat sessions with 2 very large samples of Dutch texts, the latter providing control data that are independent from a suicide prevention helpline. First, we included the CC100-Dutch dataset, a large collection of Dutch sentences (N=229,914,131), which were extracted from websites that were included in the January to December 2018 CommonCrawl snapshots from the CC-Net repository [53]. Second, we included a comparison to the language of a large sample of users on Twitter (now X), a popular microblogging platform. Using an open-access repository of COVID-19 messages [54], cohorts of active Twitter users in Dutch municipalities with more than 100,000 inhabitants in 2020 [55] were constructed based on the user-provided location information on their own profile page. Concretely, users were included who indicated that they live in one of the following cities: Amsterdam, Rotterdam, The Hague, Utrecht, Eindhoven, Groningen, Tilburg, Almere, Breda, Nijmegen, Apeldoorn, Haarlem, Arnhem, Hoofddorp, Amersfoort, Enschede, Zaanstad, Den Bosch, Zwolle, Leiden, Leeuwarden, Zoetermeer, Maastricht, Ede, Dordrecht, Alphen aan den Rijn, Alkmaar, Delft, Emmen, Venlo, and Deventer. To ensure that these Twitter users were indeed individuals, we used the M3 system [56] (a deep learning classifier trained on large collections of Twitter profiles) to classify accounts according to 3 categories: gender, age, and organization. Using the outcomes of the organization classifier, we only kept accounts that had a likelihood score of more than 0.8 of being an individual in our analysis. In total, this dataset consists of 4829 Twitter users for which the individual timelines (3200 most recent tweets, time-sorted by date posted by individual) were collected using Twitter’s application programming interface for an analytical sample of 4,600,054 tweets that were posted between July 1, 2017, and October 31, 2020. Using 0.8 as a threshold for gender and 0.6 as a threshold for age (as this category contains 4 labels rather than 2), we can assess the demographics of the Twitter users we have included in our analysis. In total, our sample contains 3119 male individuals (64 of whom were aged 18 years and younger, 117 aged 19-29 years, 455 aged 30-39 years, and 1959 aged 40 years and older) and 1522 female individuals (39 of whom were aged 18 years and younger, 145 aged 19-29 years, 202 aged 30-39 years, and 839 aged 40 years and older). Note that the totals do not necessarily have to sum up to the total amount of included Twitter users, as the outcomes of the M3 system are below the used threshold (0.8 for gender and 0.6 for age).

### Cognitive Distortions and CBT

Cognitive distortions are maladaptive patterns of thought that individuals with depression exhibit in which unrealistic and overly negative views about the world, the future, and oneself cause a cascade of detrimental cognitive, affective, and behavioral changes associated with internalizing disorders [41-43]. These cognitive distortions are frequently described in terms of 12 general types, for example, “I will never amount to anything,” which in this case amounts to “fortune-telling,” “dichotomization,” and “personalizing.” Regardless of type, all cognitive distortions share the characteristic that they amount to unrealistic, exaggerated, and overly rigid thinking patterns.

CBT, now the leading treatment of depression, anxiety, and suicide [44-49], considers cognitive distortions an important intervention target. In fact, the very effectiveness of CBT demonstrates the importance of the identification and mitigation of cognitive distortions in an individual seeking help with depression or anxiety.

### Construction of Set of Cognitive Distortion Schemata N-Grams

The detection of cognitive distortions in written language, in open-form online chat sessions, remains an open question. Some recent advances suggest natural language processing and machine learning can be leveraged to detect the presence of cognitive distortions in text [57,58] at reasonable levels of accuracy, even matching the performance of human raters, but it is not clear how well these methods generalize to online chat data, nor whether the lexical features involved in the classification correspond to our understanding of cognitive distortions [59]. In fact, some research indicates that sophisticated machine-learning models, such as deep learning, may in fact be outperformed by more transparent models that capture a small number of specific expressions associated with cognitive distortions [60].

Here, we used a theory-driven method proposed by Bathina et al [50], namely a lexicon of 241 one- to five-grams that was specifically designed and vetted by a panel of experts in CBT, originally introduced by Beck [41-43], to capture the sequences of words involved with the expression of 12 common types of cognitive distortions (see Table 1). For example, the 1-gram “never,” among other 1-grams, is deemed to be indicative of “dichotomization,” and the 3-grams “they will think” or “I am a” are deemed to be indicative of “mindreading” and “labeling,” respectively. A message that contains either one of the n-grams in the cognitive distortion schemata (CDS) lexicon is deemed to contain an indication of the expression of the specific cognitive distortion type, regardless of its topic or context. For example, the message “I will never be loved” contains the 3-gram “I will never,” which is associated with the expression of a cognitive distortion of the “fortune telling” type. Counting the number of matches of the entire CDS lexicon against the content of the chat-session messages (by help seekers or counselors) provides an estimate of the prevalence of cognitive distortion expressions in their language.

**Table 1.** Descriptive statistics of our set of cognitive distortion schemata (CDS), which are grouped in 12 common types (“distortion type”)^a^.

Distortion type	Notation	*N*_CDS_	*N*_∃_	*N*_regex_
*Catastrophizing*	*c*	25	16	15
*Dichotomous reasoning*	*dr*	25	24	4
*Disqualifying the positive*	*dtp*	14	13	8
*Emotional reasoning*	*er*	10	10	3
*Fortune-telling*	*f*	8	5	8
*Labeling and mislabeling*	*lam*	47	45	35
*Magnification and minimization*	*mam*	9	9	2
*Mental filtering*	*mf*	16	15	10
*Mindreading*	*m*	72	63	63
*Overgeneralizing*	*o*	20	20	12
*Personalizing*	*p*	15	15	7
*Should statements*	*ss*	4	4	4
Total	ℂ	265	239	171

a The column “*N*_CDS_” indicates the number of schemata in the specific category, the column “*N*_∃_” shows the number of n-grams in each category that were actually found in the chat session data, and the column “*N*_regex_” indicates the number of schemata that contain a regular expression.

The CDS lexicon is grouped in 12 distortion types following a common taxonomy, but its use does not rely on claims with respect to the component structure of cognitive distortions in general. Each n-gram is matched independently. The number of literal matches of the n-grams of the CDS lexicon in the messages of either help seekers or 113 counselors is normalized by message volume to determine CDS prevalence (see section “Measuring CDS in chat messages”).

The original CDS n-grams were translated from English to Dutch by 4 bilingual native language speakers of Dutch, namely 2 Dutch-speaking coauthors, and in-house 113 helpline psychologists Wilco Janssen and Jeroen Gomes, to match the language in which the 113 helpline conversations were conducted. The resulting set of 4 translations (for each CDS) was combined to a single list of translated CDS n-grams through a process of iterative design. Finally, 2 Dutch experts in CBT (Claudi Bockting and Marcus Huibers) were asked to validate whether the translations still captured the distorted thinking.

The translations focused on retaining the distorted thinking aspect of the schemata, respecting grammar (eg, gender) and idiomatic expressions. For that reason, the number of Dutch schemata (N=265) does not equal the number of English schemata (N=241), since one-on-one translation was not always feasible. Moreover, due to differences in Dutch word ordering (eg, verb at the end of the sentence), many of the schemata had to be formulated as regular expressions instead of n-grams. The number of schemata per category in the Dutch CDS set along with the number of observed schemata per category can be found in Table 1. The complete set of CDS is freely available in Table 2.

**Table 2.** Cognitive distortion types and the corresponding schemata in Dutch.

Distortion type	Cognitive distortion schemata
Catastrophizing	(zal|gaat)(.+ |)falen, zal(.+ |)fout gaan, zal(.+ |)verkeerd gaan, zal(.+ |)mis gaan, (zal|gaat)(.+ |)mislukken, (zal|gaat)(.+ |)eindigen, (zal|gaat)(.+ |)stoppen, (zal|gaat)(.+ |)ophouden, wordt onmogelijk, zal onmogelijk zijn, (zal|gaat) niet (gebeuren|plaatsvinden), wordt vreselijk, zal vreselijk zijn, wordt verschrikkelijk, zal verschrikkelijk zijn, wordt een ramp, zal(.+ |)een ramp zijn, (zal|gaat) nooit (eindigen|stoppen), (stopt|eindigt) niet, houdt niet op, gaat niet (stoppen|ophouden)
Dichotomous reasoning	alleen, elke, iedere, iedereen, allemaal, alles, overal, altijd, perfect, ideaal, het (aller|)?beste?", alle, niet (één|een), geen enkele, niemand, geen een, niets, niks, nergens, nooit, waardeloos, het (aller|)?ergste?, noch, (of|ofwel)(.+ |)of, zwart[-/]wit, zwart of wit, ooit
Disqualifying the positive	top,? maar, geweldig,? maar, goed,? maar, ok(e|é)?,? maar, niet echt (geweldig|top), niet zo goed, het was niet, niet zo, prima,? maar, acceptabel,? maar, afdoende,? maar
Emotional reasoning	maar ik voel, maar ik heb het gevoel, want ik voel, want ik heb het gevoel, omdat ik voel, omdat ik heb het gevoel, maar het(.+ |)voelt, want het(.+ |)voelt, omdat het(.+ |)voelt, voelt nog steeds
Fortune-telling	ik zal(.+ |) niet, we zullen(.+ |) niet, j(ij|e) (zal|zult)(.+ |) niet, z(ij|e) zullen(.+ |) niet, het zal(.+ |)niet, dat zal(.+ |)niet, hij zal(.+ |)niet, z(e|ij) zal(.+ |)niet
Labeling and mislabeling	ik ben een, hij is een, z(e|ij) is een, z(e|ij) zijn een, (het|'t) is een, d(a|i)t is een, slecht in, waardeloos voor, ik(.+ |)nooit, hij(.+ |)nooit, z(e|ij)(.+ |)nooit, w(e|ij)(.+ |)nooit, j(e|ij)(.+ |)nooit, (een|tot) last, een comple(et|te), een tota(al|le), een enorme?, een gro(te|ot), een loser, een mislukking, een zwak(ke)?, een absolu(ut|te), een volslagen, een slechte, een gebroken, een kapotte, een beschadigde?, een hulpelo(os|ze), een hopelo(os|ze), een incompetente?, een onbekwa(am|me), een giftige?, een toxische?, een lelijke?, een ongewenste?, een onbegeerlijke?, een niet geliefd, een ongeliefde?, een waardelo(os|ze), een vreselijke?, een afschuwelijke?, een verschrikkelijke?
Magnification and minimization	slechtste?, beste?, niet belangrijk, niet relevant, irrelevant, telt niet, onbelangrijk, maakt niet uit, het enige
Mental filtering	ik zie alleen, ik zie slechts, het enige (w|d)at ik zie, kan alleen(maar)? denken, (niks|niets) goeds?, (niks|niets) juist, (compleet|heel) slecht, (compleet|heel) (verkeerd|fout), alleen het (slechte|verkeerde), alleen het slechtste, alleen het ergste, (als|kon) ik maar, ik wou dat ik, als ik nou, (als|kon) het maar, als het nou
Mindreading	iedereen gelooft, iedereen weet, iedereen denkt, iedereen zal(.+ |)geloven, iedereen zal(.+ |)weten, iedereen zal(.+ |)denken, niemand gelooft, niemand weet, niemand denkt, niemand zal(.+ |)geloven, niemand zal(.+ |)weten, niemand zal(.+ |)denken, hij gelooft, hij weet, hij denkt, hij gelooft(.+ |)niet, hij weet(.+ |)niet, hij denkt(.+ |)niet, hij zal(.+ |)geloven, hij zal(.+ |)weten, hij zal(.+ |)denken, hij zal(.+ |)niet geloven, hij zal(.+ |)niet weten, hij zal(.+ |)niet denken, z(ij|e) gelooft, z(ij|e) weet, z(ij|e) denkt, z(ij|e) gelooft(.+ |)niet, z(ij|e) weet(.+ |)niet, z(ij|e) denkt(.+ |)niet, z(ij|e) zal(.+ |)geloven, z(ij|e) zal(.+ |)weten, z(ij|e) zal(.+ |)denken, z(ij|e) zal(.+ |)niet geloven, z(ij|e) zal(.+ |)niet weten, z(ij|e) zal(.+ |)niet denken, z(ij|e) geloven, z(ij|e) weten, z(ij|e) denken, z(ij|e) geloven(.+ |)niet, z(ij|e) weten(.+ |)niet, z(ij|e) denken(.+ |)niet, z(ij|e) zullen(.+ |)geloven, z(ij|e) zullen(.+ |)weten, z(ij|e) zullen(.+ |)denken, z(ij|e) zullen(.+ |)niet geloven, z(ij|e) zullen(.+ |)niet weten, z(ij|e) zullen(.+ |)niet denken, w(ij|e) geloven, w(ij|e) weten, w(ij|e) denken, w(ij|e) geloven(.+ |)niet, w(ij|e) weten(.+ |)niet, w(ij|e) denken(.+ |)niet, w(ij|e) zullen(.+ |)geloven, w(ij|e) zullen(.+ |)weten, w(ij|e) zullen(.+ |)denken, w(ij|e) zullen(.+ |)niet geloven, w(ij|e) zullen(.+ |)niet weten, w(ij|e) zullen(.+ |)niet denken, j(ij|e) gelooft, j(ij|e) weet, j(ij|e) denkt, j(ij|e) gelooft(.+ |)niet, j(ij|e) weet(.+ |)niet, j(ij|e) denkt(.+ |)niet, j(ij|e) (zal|zult)(.+ |)geloven, j(ij|e) (zal|zult)(.+ |)weten, j(ij|e) (zal|zult)(.+ |)denken, j(ij|e) (zal|zult)(.+ |)niet geloven, j(ij|e) (zal|zult)(.+ |)niet weten, j(ij|e) (zal|zult)(.+ |)niet denken
Overgeneralizing	de hele tijd, gebeurt altijd, (steeds|altijd) zo, gebeurt (elke|iedere) keer, compleet”, "helemaal, niemand(.+ |)ooit, z(ij|e)(.+ |)allemaal, jullie(.+ |)allemaal, ik(.+ |)altijd, j(ij|e)(.+ |)altijd, hij(.+ |)altijd, z(ij|e)(.+ |)altijd, ik ben altijd, j(ij|e) bent altijd, hij is altijd, z(ij|e) is altijd, z(ij|e) zijn altijd
Personalizing	(he|al)lemaal mijn, omdat ik, omdat mijn, (door|vanwege) mijn, (door|vanwege) mij, ik ben verantwoordelijk, geef mij de schuld, mij de schuld geven, ik veroorzaakte, ik heb(.+ |)veroorzaakt, ik voel m(ij|e) verantwoordelijk, (he|al)lemaal door mij, allemaal mijn schuld, mijn (fout|schuld), mijn verantwoordelijkheid
Should statements	zou, zouden, moet, moeten

### Measuring CDS in Chat Messages

Let *S* denote the set of all chat sessions. Each chat session *s*∈*S* in our sample set consists of a collection of *k* time-ordered messages, *s*={*m*_1_, *m*_2_, ..., *m_k_*}. Each message *m_i_* in the session is marked by an indicator of the role of who posted that message denoted by $r_{m_{i}}$, that is, counselor, help seeker, or system, denoted by *o*, *h*, or *b*, respectively, and the time at which the message was posted (in s from the start of the session), denoted by $t_{m_{i}}$.

Additionally, as mentioned, we have defined a set of 265 Dutch CDS n-grams, denoted as *C*. Each CDS, denoted as *c*, is intended to represent the lexical building blocks of expressing cognitive distortions. The notation for the collection of CDS within a distortion type is indicated in the column “Notation” of Table 1 (eg, ${\mathbb{C}}_{c}$ denotes all CDS belonging to the category *Catastrophizing*). We define a CDS matching function *g_C_*(*m_i_*)→{0,1}, which assigns each message *m_i_* to 0 or 1 based on whether that message contains at least 1 CDS that is contained in the set C⊆C.

### Calculating CDS Prevalence

As we are interested in the prevalence of cognitive distortions in messages, we measured the fraction of messages posted that contain a CDS. Thus, we calculated the CDS prevalence for a given set of CDS *C*, a role *r* (either help seeker or counselor), and a set of chat session *S* as the ratio of messages that contain a CDS marker over the total number of messages.

For the NL cohort, *S* can be seen as the set of timelines of the individuals in the cohort, in which *s*∈*S* represents the timeline of an individual that consists of a collection of tweets rather than messages. Finally, for the NL web dataset, *S* consists of a single element, that is, a collection of sentences rather than messages.

### Calculating CDS Prevalence Ratio

We calculated the prevalence ratio between roles as follows:

.$$R_{C}(r_{1},r_{2},S)=\frac{P_{C}(r_{1},S)}{P_{C}(r_{2},S)}$$

## Results

Our analysis of CDS prevalence in the language of 113 help seekers was not diachronic, that is, we compared CDS prevalence for all messages written by a specific cohort regardless of when the messages were recorded. Therefore, we assessed whether message volume and CDS prevalence remained stable over the time period of our analysis to avoid any bias resulting from our choice of time period. The messages exchanged in the 113 service were recorded in a time interval that spans almost 4 years, roughly from 2017 to 2021, during which changes in both message volume (possibly reflecting lesser or greater prevalence of suicidality) and the nature of the calls (seasonal or topical variation) may have occurred. In particular, the last 2 years of the analysis period include the COVID-19 pandemic (declared a pandemic in March 2020).

**Figure 1.**
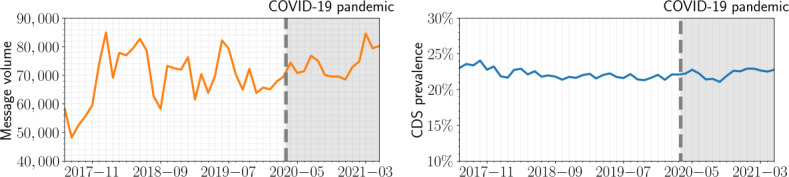
Message volume (number of messages, left panel) and cognitive distortion schemata (CDS) prevalence (%, right panel) for the help seeker cohort over the time period of analysis. The vertical line marks the start of the COVID-19 pandemic in March 2020. No significant changes in message volume nor CDS prevalence (%) were observed for the time period under consideration, including before or after the start of the COVID-19 pandemic.

As shown in Figure 1, we observed only slight changes in the volume of monthly messages from July 2017 to June 2021; the median monthly message volume is 70,768 (IQR 11,207), and we saw no indications of discontinuities, interruptions, or other changes associated with the start of the COVID-19 pandemic, nor any significant seasonal trends. Importantly, the monthly CDS prevalence (the ratio of monthly CDS matches in messages over the monthly number of messages) showed even less variation, fluctuating in a narrow range around 22.2% (95% CI 21.3-23.5), indicating no significant changes in CDS prevalence over time; hence, there is no indication of topical changes related to the COVID-19 pandemic. The monthly CDS prevalence separated by the CDS type was equally stable over time (see Figure S1 in Multimedia Appendix 1).

Having verified the absence of significant trends, seasonal changes of message volume nor CDS prevalence over the time period of our analysis, we turned to comparing the cohort-level CDS prevalence between help seekers and counselors across the entire time range. The counselors were used as a control since they are expected to be least affected by suicidality and depression, yet are conversing with help seekers on the same platform, about the same general topic, and in the same language (Dutch).

The overall CDS prevalence between messages entered by both help seekers and counselors, respectively (across the entire time period), showed a pronounced difference of 22.19% and 13.78%, which is mostly concentrated in some CDS types (see Table 1 for denotations), that is, *dichotomous reasoning* (*P_dr_*(*h*)=0.13 and *P_dr_*(*o*)=0.07), *should statements* (*P_ss_*(*h*)=0.05 and *P_ss_*(*o*)=0.02), and *overgeneralizing* (*P_o_*(*h*)=0.03 and *P_o_*(*o*)=0.02), for the help seeker and counselor cohorts separately, indicating CDS type–specific effects.

**Figure 2.**
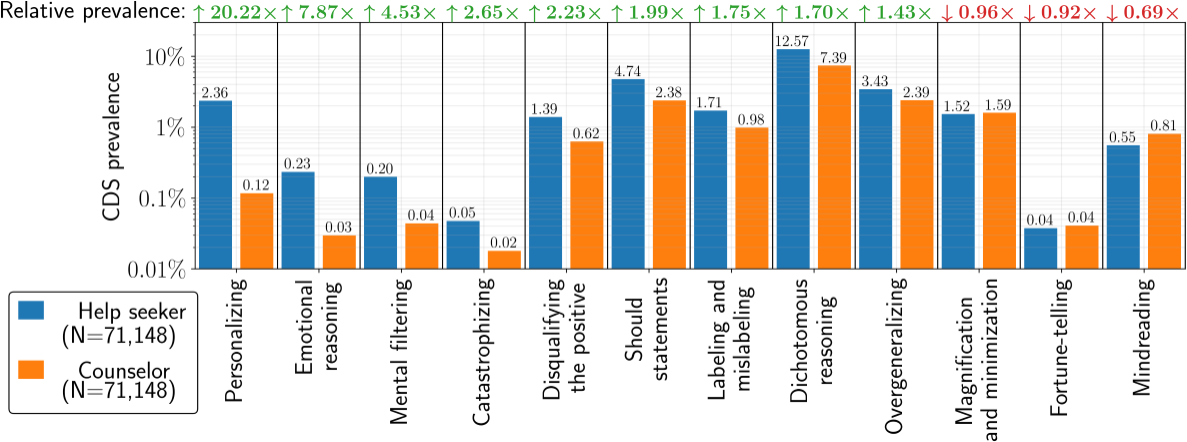
Cognitive distortion schemata (CDS) prevalence for the help seeker and counselor cohorts stratified per category. CDs types are ranked left to right by the magnitude of CDS prevalence ratio. Each set of 2 bars indicates the CDS prevalence *P_C_* for the type *C*, separated by the help seeker cohort (*P_C_*(h), blue) and the counselor cohort (*P_C_*(*o*), orange), respectively. The prevalence ratio (*$R_{C}\text{=}\frac{P_{C}\left(h\right)}{P_{C}\left(o\right)}$*) for each category *C* is listed at the top with up-and-down arrows indicating whether the CDS of a particular category is either more or less prevalent in the help seeker cohort than the counselor cohort for that category.

Based on a comparison of CDS prevalence, overall or separated by the CDS type, help seekers exhibited higher CDS prevalence in their language than 113 counselors. To determine the magnitude of this effect by the CDS type, we calculated the *CDS prevalence ratio* by the CDS type between the help seeker and counselor cohorts (CDS prevalence ratio $R\text{=}\frac{P\left(h\right)}{P\left(o\right)}$) as shown in Figure 2. The prevalence ratio between the *h* and *o* cohorts can be separated by the CDS type; for example, for CDS *personalizing* types, we can calculate the CDS prevalence ratio between the help seeker and counselor cohorts *R*_*p*_.

As shown in Figure 2, we found that although CDS prevalence is strongly concentrated in some CDS types, the CDS prevalence in the help seeker cohort was significantly larger than CDS prevalence in the counselor cohort for nearly all 12 CDS types, with the exception of “magnification and minimization,” “fortune telling,” and “mindreading.”

In fact, CDS prevalence was 20 times higher in help seeker messages than those of 113 counselors for the *Personalizing* types of CDS; *R*_*p*_=20.22×, followed by *emotional reasoning* (*R*_*er*_=7.87×), *mental filtering* (*R*_*p*_=4.53×), *catastrophizing* (*R*_*c*_=2.65×), and *disqualifying the positive* (*R*_*dtp*_=2.23×).

In addition to the analysis of the differences between the 2 cohorts, we extended our analysis by investigating the difference in CDS prevalence at the level of individual chat sessions. For this analysis, we determined the prevalence of CDS phrases across the messages in a singular chat session for each role (ie, help seeker and counselor) and subsequently analyzed the distribution of this quantity across all chat sessions in the dataset. This analysis sheds more light on interpersonal differences between the individuals across both considered cohorts. Based on this analysis at the level of chat sessions, we found that the mean values of *within*-session CDS prevalence for help seeker and counselor cohorts are 0.228 (SD 0.119) and 0.135 (SD 0.075), respectively. A Bartlett test (*T*=14,581, *P*<.001) indicated that the CDS prevalence SDs are different for the help seeker and the counselor cohorts. We therefore used the Welch *t* test to find that CDS prevalence was different across the help seeker and counselor cohorts (*t*_120,048_=177, *P*<.001, *d*=0.94).

The language of 113 counselors may vary significantly from that of the general population in an online chat environment and could be expected to have exceptionally low levels of CDS prevalence relative to the general population, given their training and professional role. Therefore, we also included a comparison with the observed *within*-individual CDS prevalence of a cohort of Twitter users in the NL cohort and a collection of Dutch sentences extracted from the web.

The first was a comparison to the language of a large sample of Twitter (now X) users, since Twitter as a microblogging platform was specifically designed for the communication of short, chat-like messages, not dissimilar in format and style from an online chat environment. Using a similar approach to the study by Bathina et al [50], we established a cohort of Twitter users who indicated on their user profile that they lived in Dutch municipalities with more than 100,000 inhabitants in 2020 [55], which we refer to as the NL cohort. For each of the 4829 individuals in this NL cohort, we retrieved a record of up to 3200 of their most recent Tweets, which were subjected to the same CDS prevalence analysis as the messages and sessions of 113 help seekers and counselors.

The second was a comparison with a large corpus of general Dutch text observed online using the January to December 2018 Commoncrawl snapshots from the CC-Net repository [53], referred to as the NL web dataset. This snapshot contains a large collection of Dutch sentences (N=229,914,131), called the CC100-Dutch dataset.

**Figure 3.**
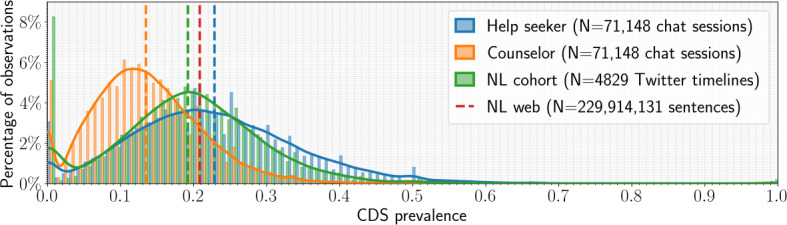
Within-session cognitive distortion schemata (CDS) prevalence distributions for the help seeker and counselor cohorts (N=71,148 chat sessions), the NL cohort (N=4829 Twitter timelines), and the mean CDS prevalence in the NL web dataset (N=229,914,131 sentences). The panel on the left displays the kernel density estimate based on the observations in each cohort. The 3 panels on the right display the distributions of the observations in each cohort. The dashed vertical line indicates the mean CDS prevalence.

The mean *within-individual* CDS prevalence for the Twitter NL cohort is 0.192 (SD 0.107), meaning that 19.2% of the tweets of the individuals in the NL cohort contain a CDS. Moreover, 20.8% of the Dutch sentences in the NL web dataset contain a CDS. The overall distribution of CDS prevalence for both the chat sessions and *Twitter* timelines is shown in Figure 3. We compared the CDS prevalence across these cohorts with Bartlett and Welch *t* tests, adjusting for multiple comparisons with a Bonferroni correction, the results of which are shown in Table 3.

**Table 3.** Comparing the variance (Bartlett *t* test) and mean (Welch *t* test) of cognitive distortion schemata (CDS) prevalence distributions for (1) help seekers, (2) 113 counselors, and (3) individuals living in the Netherlands on Twitter^a^.

Cohort comparison	Bartlett test		Welch *t* test		
	*T*	*P* value	*t* (*df*)	*P* value	Cohen *d*
Help seeker versus counselor	14,581	<.001	177 (120,048)	<.001	0.94
Help seeker versus NL cohort	93	<.001	22.6 (5667)	<.001	0.031
Counselor versus NL cohort	1415	<.001	−36.7 (5155)	<.001	0.74

a To account for multiple hypothesis testing, we applied a Bonferroni correction, that is, α=0.05/3 to determine significance levels.

For all comparisons, we could reject the null-hypothesis at the α level given by the Bonferroni correction. The relative differences indicated that the counselor cohort exhibits the lowest CDS prevalence, possibly because of their training and their role as providers of assistance in exchanges with individuals seeking help with suicidality. Although the mean values of CDS prevalence for the NL web, the NL cohort, and the help seeker cohort were very close to each other when examining the distributions, the help seeker cohort had a greater likelihood of individuals with a high CDS prevalence than the NL cohort.

## Discussion

### Principal Results

We employed a theory-driven approach to investigate the prevalence of expressions of cognitive distortions, that is, maladaptive thought patterns that characterize internalizing disorders, such as depression and anxiety [41-43], in a large collection of chat sessions of a suicide prevention helpline in the NL between help seekers and counselors and 2 baselines extracted from large-scale corpora of Dutch social media and web content.

Our analysis investigated the CDS prevalence in the recorded chat sessions of the Dutch suicide prevention helpline. Our aim was to quantify the levels of cognitive distortions in the language of online help seekers versus 2 controls that include the 113 respondents. Given the comorbidity between suicide and depression [36-38], and the fact that the latter is associated with cognitive distortions [41-43], we expected high levels of cognitive distortions in the language of those who contact suicide helplines.

The monthly CDS prevalence was quite stable during the entire observation period, even during the 2 years of a devastating worldwide pandemic, which according to some analyses led to topical changes in Dutch suicide helpline conversations (eg, an 808% increase of the topic of “COVID-19”) [61]. This shows that even though the topics that are discussed in the chats change over time, the degree of distorted expressions does not seem to change. This is surprising given the backdrop of a global pandemic. Future work may explore the stability of CDS prevalence across time in online environments further. There are some indications of a minor trend toward increased session volume starting in early 2021. However, this does not seem to affect the monthly CDS prevalence significantly, which is in line with other research studies that showed that the prevalence of high anxiety and depression symptom levels did not increase [62]. This is the case across all separate CDS types (see Figure S1 in Multimedia Appendix 1).

In general, certain types of CDS n-grams are more prevalent than others; the CDS prevalence for *fortune-telling* remained low at 0.04%, while the CDS prevalence for *dichotomous reasoning* was 2 orders of magnitude more prevalent at 12.57% across all chat sessions, as indicated in Figure 2. In general, categories that relate to black-and-white thinking (eg, *dichotomous reasoning* and *overgeneralizing*) were most frequent among both help seekers and counselors, whereas categories that relate to emotion and affect (eg, *catastrophizing*, *emotional reasoning*, and *disqualifying the positive*) were less frequent. This could reflect how some distortion types are generally more or less common in Dutch or how CDS n-grams of a specific type are worded in the lexicon. In fact, many n-grams contain some of the most frequent Dutch words (eg, “ik” (I) or “ben” (am)), since the CDS lexicon was designed to capture common, context-free patterns of language. It is expected that shorter n-grams occur more frequently in the language than longer n-terms.

We compared the CDS prevalence ratio between help seekers and counselors, since the latter is least likely to be affected by suicidality and depression. We observe that nearly all types of cognitive distortions are significantly more prevalent in help seeker messages than those of the 113 counselors. CDS of the *personalizing*, *emotional reasoning*, and *mental filtering* types are, respectively, 20.22, 7.87, and 4.53 times more prevalent in the help seeker cohort than the counselor cohorts. In fact, 5 categories are used at least twice as frequently in the help seeker cohort as in the counselor cohort, that is, *personalizing*, *emotional reasoning*, *mental filtering*, *catastrophizing*, and *disqualifying the positive*. The fact that the differences for other categories are less pronounced could be explained by counselors mimicking the phrasings of help seekers in their chat in their attempt to build connection with the help seeker, a phenomenon called lexical entrainment [63]. The CDS n-grams have in previous research been shown not to have significant valence loadings [50] nor is their prevalence in the language of individuals with depression driven by the presence of personal pronouns. Hence, the expected higher level of negativity or self-referential nature of the help seekers' messages is not likely to explain the higher prevalence of the set of CDS n-grams in the language of help seekers.

Our analysis of the *within*-session CDS prevalence shows clear differences in CDS prevalence between the help seeker and counselor cohorts, with the help seeker cohort having significantly a higher mean CDS prevalence than the counselor cohort. This may be expected because the counselor cohort has been professionally trained to communicate with help seekers, which possibly included the avoidance of cognitive distortions in their communications with help seekers. To verify the degree to which counselors exhibit exceptionally low levels of cognitive distortions in their language, we compared the prevalence of CDS in their language and that of the help seekers to a cohort of Dutch individuals (referred to as the NL cohort) on Twitter, since the latter as the microblogging platform may closely approximate the format and vernacular of online chat helplines.

As expected, the counselor cohort has significantly a lower mean CDS prevalence than the NL cohort, that is, their language contains fewer markers of cognitive distortions than a random sample of Dutch Twitter users. As mentioned, this lower usage of CDS in the counselor cohort is likely a result of the training they have received in handling these chat sessions at 113. Another fact that stands out in Figure 3 is the difference in variability between the help seeker and the counselor cohorts, which indicates that there are significant differences between individual chat sessions and that the 113 service is reaching a wide audience of individuals with varying degrees of urgency and the need of assistance. These results highlight the usability of the CDS annotations in the chat sessions to further analyze these conversations, for example, by providing the counselors with real-time feedback during chat sessions, or by highlighting important changes in language during the sessions that could give an indication of the perceived quality of the conversation.

Our data were collected by an anonymous helpline, which in the context of providing urgent, low-threshold care did not collect information on neurodevelopmental diagnoses. Certain neurodevelopmental conditions have been associated with differences in cognitive styles, which may relate to variations in the expression of cognitive distortions. Additionally, some neurodivergent populations have been shown to experience higher levels of suicidal ideation and suicide attempts [64,65]. However, the relationship between such differences and the prevalence of cognitive distortions remains insufficiently characterized. Future research should examine how these individual factors may interact with the prevalence of cognitive distortions in the context of suicide helplines.

Nevertheless, we have converging evidence across 4 different online samples (113 help seekers, 113 counselors, a large cohort of random Dutch Twitter users, and a large collection of Dutch online texts), indicating that the prevalence of cognitive distortion markers in the language of 113 help seekers is exceptionally high.

### Limitations

Several limitations of our approach should be considered.

#### Constructing a Good Baseline for the 113 Chat Platform Is Challenging

It is difficult to gauge the magnitude of CDS prevalence in the language of the help seekers in practice without a proper baseline or counterfactual. Our sample does not include individuals contacting the same helpline without suicidal ideation or intent while the 113 counselors are trained professionals who may avoid expressions of cognitive distortions in their language or responses to help seekers. Although the format and vernacular of messages on Twitter as a microblogging platform may approximate that of chat environments, it may still fall short in when compared to the 113 chat platform. For instance, the focus of a microblogging platform is not on having conversations as opposed to a helpline, nor will it discuss the same topics.

#### CDS Annotations Do Not Necessarily Equate to Cognitive Distortion

We emphasize that not all uses of CDS n-grams reflect actual cognitive distortions. Although the set of CDS n-grams was specifically designed to capture the expression of common types of cognitive distortions by experts in CBT and shown to capture differences in the language between individuals with depression and a random sample, these phrases can be part of nondepressed expressions, possibly leading to false positives. However, such false positives would be equally likely in either one of the examined cohorts. Furthermore, the CDS lexicon does not exhaustively cover all possible expressions of cognitive distortions in the language as it contains 241 n-grams. This may cause false negatives where a cognitive distortion may have been expressed in a statement but was left unrecognized by the CDS lexicon. On balance, the presence of a CDS does not provide proof positive evidence of the presence of maladaptive thought patterns, but consistently observing CDS prevalence across cohorts allows for a fair comparison between cohorts. Future work should explore the validity of CDS occurrence as an indicator of cognitive distortions by comparing them to human annotations.

#### Cognitive Distortions Do Not Necessarily Map One-to-One to Specific Categories

The CDS n-grams are designed to represent 1 type of cognitive distortion among 12 commonly discussed types in the literature. However, in practice, CDS n-grams of different types can occur in the same message and reflect overlapping types of cognitive distortions. Furthermore, the specific types discussed in the literature are generally used to illustrate the concept of cognitive distortions, without necessarily making claims about the component structure of the concept of cognitive distortions, which may rather all belong to the same class of rigid, unrealistic, and exaggerated thinking. As a result, the observed CDS prevalence by category likely underestimates true prevalence. Based on the distribution of *within*-session CDS prevalence of both cohorts, this underestimation reduces the relative difference in the CDS prevalence of a given category, reducing the observed effects instead of exaggerating them.

### Ethical Reflections

Given the sensitive nature of both the data and the possible applications of our considered technique, there are several ethical reflections that we want to address explicitly. First, given the sensitive nature of the content of the chat sessions, all data were processed in the aggregate, without personal identifiers, ensuring the anonymity of all analyses and research outcomes. Next, we stress that our aim is not to diagnose individuals with cognitive distortions or suicidality nor is it our intention to underpin replacements of human counselors by automated chatbots [66,67]. Based on our current results, we believe that our research has the potential to provide additional guidance or support to the counselors of the suicide prevention helpline by highlighting important changes in language that are known to be implicated in the development and recovery trajectories of internalizing disorders. However, careful considerations must be made before such techniques are put into practice [67,68]. Future work will focus on more accurate methods to detect cognitive distortions in language, such as the use of more sophisticated natural language processing and artificial intelligence, including ground truth obtained from human raters. Our analysis did not investigate changes in language over the course of a chat session as the conversation between the help seeker and the counselor unfolds, which may provide indicators of the effectiveness of the exchange. Future research should examine whether the changes of CDS prevalence during a conversation, in subsequent utterances, may shape its perceived quality and effectiveness.

### Conclusions

We presented converging evidence across 4 different online samples (113 help seekers, 113 counselors, a large cohort of random Dutch Twitter users, and a large collection of Dutch online texts), indicating that the prevalence of cognitive distortion markers in the language of 113 help seekers is exceptionally high. Our results point to the value of lexical analysis as an instrument to detect the cognitive and lexical markers of suicidality.

## Supplementary material

10.2196/81213 Multimedia Appendix 1 The variations in frequency and prevalence of CDS markers in both the help seeker and operator cohorts. Moreover, it presents an analysis that compares different types of help seekers (individuals with suicide ideation vs friends/next of kin) that reach out to the 113 helpline as a control group.

## Abbreviations

CBT: cognitive behavioral therapy
CDC: Centers for Disease Control and Prevention
CDS: cognitive distortion schemata
WHO: World Health Organization

## References

[1] (2021). Suicide worldwide in 2019: global health estimates. https://iris.who.int/server/api/core/bitstreams/3bd4ac79-4347-420e-b675-948d36ab3d90/content.

[2] Fanslow J, Coggan C, Miller B, Norton R (1997). The economic cost of homicide in New Zealand. Soc Sci Med.

[3] Corso PS, Mercy JA, Simon TR, Finkelstein EA, Miller TR (2007). Medical costs and productivity losses due to interpersonal and self-directed violence in the United States. Am J Prev Med.

[4] Kennelly B (2007). The economic cost of suicide in Ireland. Crisis.

[5] Kinchin I, Doran CM (2018). The cost of youth suicide in Australia. Int J Environ Res Public Health.

[6] Shepard DS, Gurewich D, Lwin AK, Reed GA, Silverman MM (2016). Suicide and suicidal attempts in the United States: costs and policy implications. Suicide & Life Threat Behav.

[7] Pil L, Pauwels K, Muijzers E, Portzky G, Annemans L (2013). Cost-effectiveness of a helpline for suicide prevention. J Telemed Telecare.

[8] Looijmans M, Elzinga E, Popma A, van Bergen D, Gilissen R, Mérelle S (2024). Understanding the needs and perspectives of young adults with recent suicidal ideation: insights for suicide prevention. Front Child Adolesc Psychiatry.

[9] (2019). Key substance use and mental health indicators in the United States: results from the 2018 National Survey on Drug Use and Health. https://mamh-web.files.svdcdn.com/production/files/NSDUHNationalFindingsReport2018.pdf?dm=1615222948.

[10] Ivey-Stephenson AZ, Demissie Z, Crosby AE (2020). Suicidal ideation and behaviors among high school students - Youth Risk Behavior Survey, United States, 2019. MMWR Suppl.

[11] Patel V, Saxena S, Lund C (2018). The Lancet commission on global mental health and sustainable development. The Lancet.

[12] Mokkenstorm JK, Eikelenboom M, Huisman A (2017). Evaluation of the 113 online suicide prevention crisis chat service: outcomes, helper behaviors and comparison to telephone hotlines. Suicide Life Threat Behav.

[13] Hoffberg AS, Stearns-Yoder KA, Brenner LA (2019). The effectiveness of crisis line services: a systematic review. Front Public Health.

[14] Zalsman G, Hawton K, Wasserman D (2016). Suicide prevention strategies revisited: 10-year systematic review. Lancet Psychiatry.

[15] Sindahl TN, Côte LP, Dargis L, Mishara BL, Bechmann Jensen T (2019). Texting for help: processes and impact of text counseling with children and youth with suicide ideation. Suicide Life Threat Behav.

[16] Mazzer K, O’Riordan M, Woodward A, Rickwood D (2021). A systematic review of user expectations and outcomes of crisis support services. Crisis.

[17] Mathieu SL, Uddin R, Brady M (2021). Systematic review: the state of research into youth helplines. J Am Acad Child Adolesc Psychiatry.

[18] Brody C, Star A, Tran J (2020). Chat-based hotlines for health promotion: a systematic review. Mhealth.

[19] Gould MS, Chowdhury S, Lake AM (2021). National Suicide Prevention Lifeline crisis chat interventions: evaluation of chatters’ perceptions of effectiveness. Suicide Life Threat Behav.

[20] Pauwels K, De Jaegere E, Vanderreydt P, Aerts S, Vande Gaer E, Portzky G (2025). Assessing a suicide prevention helpline’s impact on caller crisis level and suicidality. Arch Suicide Res.

[21] O’Dea B, Larsen ME, Batterham PJ, Calear AL, Christensen H (2017). A linguistic analysis of suicide-related Twitter posts. Crisis.

[22] Lao C, Lane J, Suominen H (2022). Analyzing suicide risk from linguistic features in social media: evaluation study. JMIR Form Res.

[23] Yeskuatov E, Chua SL, Foo LK (2024). Detecting suicidal ideations in online forums with textual and psycholinguistic features. Appl Sci (Basel).

[24] Ji S, Yu CP, Fung S fu, Pan S, Long G (2018). Supervised learning for suicidal ideation detection in online user content. Complexity.

[25] Wiltsey Stirman S, Pennebaker JW (2001). Word use in the poetry of suicidal and nonsuicidal poets. Psychosom Med.

[26] De Choudhury M, Kiciman E, Dredze M, Coppersmith G, Kumar M Discovering shifts to suicidal ideation from mental health content in social media. https://dl.acm.org/doi/abs/10.1145/2858036.2858207.

[27] De Choudhury M, Kiciman E (2017). The language of social support in social media and its effect on suicidal ideation risk. Proc Int AAAI Conf Weblogs Soc Media.

[28] Feldhege J, Wolf M, Moessner M, Bauer S (2023). Psycholinguistic changes in the communication of adolescent users in a suicidal ideation online community during the COVID-19 pandemic. Eur Child Adolesc Psychiatry.

[29] Egnoto MJ, Griffin DJ (2016). Analyzing language in suicide notes and legacy tokens. Crisis.

[30] Teixeira AS, Talaga S, Swanson TJ, Stella M (2021). Revealing semantic and emotional structure of suicide notes with cognitive network science. Sci Rep.

[31] Leavitt J, Hong JH, Walker RL (2021). Paradoxical positivity: suicide notes use less distressed language than blogs about depression, suicidal thoughts, and even cooking. Suicide Life Threat Behav.

[32] Pennebaker JW, Boyd RL, Jordan K, Blackburn K (2015). The development and psychometric properties of LIWC2015. https://repositories.lib.utexas.edu/server/api/core/bitstreams/b0d26dcf-2391-4701-88d0-3cf50ebee697/content.

[33] Brancu M, Jobes D, Wagner BM, Greene JA, Fratto TA (2016). Are there linguistic markers of suicidal writing that can predict the course of treatment? A repeated measures longitudinal analysis. Arch Suicide Res.

[34] Salmi S, Mérelle S, Gilissen R, Brinkman WP (2021). Content-based recommender support system for counselors in a suicide prevention chat helpline: design and evaluation study. J Med Internet Res.

[35] Janssen W, van Raak J, van der Lucht Y, van Ballegooijen W, Mérelle S (2022). Can outcomes of a chat-based suicide prevention helpline be improved by training counselors in motivational interviewing? A non-randomized controlled trial. Front Digit Health.

[36] Wiebenga JXM, Dickhoff J, Mérelle SYM (2021). Prevalence, course, and determinants of suicide ideation and attempts in patients with a depressive and/or anxiety disorder: a review of NESDA findings. J Affect Disord.

[37] Bolton JM, Pagura J, Enns MW, Grant B, Sareen J (2010). A population-based longitudinal study of risk factors for suicide attempts in major depressive disorder. J Psychiatr Res.

[38] Baldessarini RJ, Tondo L, Pinna M, Nuñez N, Vázquez GH (2019). Suicidal risk factors in major affective disorders. Br J Psychiatry.

[39] Eichstaedt JC, Smith RJ, Merchant RM (2018). Facebook language predicts depression in medical records. Proc Natl Acad Sci U S A.

[40] Rude S, Gortner EM, Pennebaker J (2004). Language use of depressed and depression-vulnerable college students. Cogn Emot.

[41] Beck AT (1963). Thinking and depression. i. Idiosyncratic content and cognitive distortions. Arch Gen Psychiatry.

[42] Beck AT (1964). Thinking and depression. Arch Gen Psychiatry.

[43] Beck JS (2011). Cognitive Behavior Therapy: Basics and Beyond.

[44] Hawton K, Witt KG, Taylor Salisbury TL (2015). Interventions for self-harm in children and adolescents. Cochrane Database Syst Rev.

[45] Zhou X, Teng T, Zhang Y (2020). Comparative efficacy and acceptability of antidepressants, psychotherapies, and their combination for acute treatment of children and adolescents with depressive disorder: a systematic review and network meta-analysis. Lancet Psychiatry.

[46] Cuijpers P, Berking M, Andersson G, Quigley L, Kleiboer A, Dobson KS (2013). A meta-analysis of cognitive-behavioural therapy for adult depression, alone and in comparison with other treatments. Can J Psychiatry.

[47] Karyotaki E, Efthimiou O, Miguel C (2021). Internet-based cognitive behavioral therapy for depression: a systematic review and individual patient data network meta-analysis. JAMA Psychiatry.

[48] Lorenzo-Luaces L (2018). The evidence for cognitive behavioral therapy. JAMA.

[49] Wu H, Lu L, Qian Y (2022). The significance of cognitive-behavioral therapy on suicide: an umbrella review. J Affect Disord.

[50] Bathina KC, Ten Thij M, Lorenzo-Luaces L, Rutter LA, Bollen J (2021). Individuals with depression express more distorted thinking on social media. Nat Hum Behav.

[51] Bollen J, Ten Thij M, Breithaupt F (2021). Historical language records reveal a surge of cognitive distortions in recent decades. Proc Natl Acad Sci U S A.

[52] Salmi S, Mérelle S, Gilissen R, van der Mei R, Bhulai S (2024). The most effective interventions for classification model development to predict chat outcomes based on the conversation content in online suicide prevention chats: machine learning approach. JMIR Ment Health.

[53] Wenzek G, Lachaux MA, Conneau A, Chaudhary V, Guzmán F CCNet: extracting high quality monolingual datasets from web crawl data. https://aclanthology.org/2020.lrec-1.494/.

[54] Chen E, Lerman K, Ferrara E (2020). Tracking social media discourse about the COVID-19 pandemic: development of a public coronavirus Twitter data set. JMIR Public Health Surveill.

[55] (2020). Population on january 1st. https://opendata.cbs.nl/#/CBS/nl/dataset/70072ned/table?ts=1775640759560.

[56] Wang Z, Hale S, Adelani DI Demographic inference and representative population estimates from multilingual social media data. https://publikationen.sulb.uni-saarland.de/bitstream/20.500.11880/33357/1/p2056-wang%20%281%29.pdf.

[57] Tauscher JS, Lybarger K, Ding X (2023). Automated detection of cognitive distortions in text exchanges between clinicians and people with serious mental illness. Psychiatr Serv.

[58] Simms T, Ramstedt C, Rich M, Richards M, Martinez T, Giraud-Carrier C Detecting cognitive distortions through machine learning text analytics. https://ieeexplore.ieee.org/abstract/document/8031202.

[59] Ernala SK, Birnbaum ML, Candan KA Methodological gaps in predicting mental health states from social media: triangulating diagnostic signals. http://www.munmund.net/pubs/CHI19_MethodGaps.pdf.

[60] Shickel B, Siegel S, Heesacker M, Benton S, Rashidi P Automatic detection and classification of cognitive distortions in mental health text. https://ieeexplore.ieee.org/abstract/document/9288097.

[61] Salmi S, Mérelle S, Gilissen R, van der Mei R, Bhulai S (2022). Detecting changes in help seeker conversations on a suicide prevention helpline during the COVID- 19 pandemic: in-depth analysis using encoder representations from transformers. BMC Public Health.

[62] van der Velden PG, Contino C, Das M, van Loon P, Bosmans MWG (2020). Anxiety and depression symptoms, and lack of emotional support among the general population before and during the COVID-19 pandemic. a prospective national study on prevalence and risk factors. J Affect Disord.

[63] Brennan SE, Clark HH (1996). Conceptual pacts and lexical choice in conversation. J Exp Psychol Learn Mem Cogn.

[64] van Bentum J, Sijbrandij M, Huibers M, Begeer S (2024). Occurrence and predictors of lifetime suicidality and suicidal ideation in autistic adults. Autism.

[65] Balt E, Mérelle S, Vrinzen S (2025). Mixed-methods psychosocial autopsy study of suicide in young and middle-aged individuals in the Netherlands. BMJ Public Health.

[66] Abd-Alrazaq AA, Rababeh A, Alajlani M, Bewick BM, Househ M (2020). Effectiveness and safety of using chatbots to improve mental health: systematic review and meta-analysis. J Med Internet Res.

[67] Sedlakova J, Trachsel M (2023). Conversational artificial intelligence in psychotherapy: a new therapeutic tool or agent?. Am J Bioeth.

[68] Reddy S (2023). Evaluating large language models for use in healthcare: a framework for translational value assessment. Informatics Med Unlocked.

