# Supplementary material for: Prevalence of Cognitive Distortion Markers in a Suicide Prevention Chat Service: Mixed Methods Study
Source: JMIR Ment Health. 2026 Apr 28;13:e81213. doi: 10.2196/81213 (PMC13124084; doi:10.2196/81213)
Supplement: Multimedia Appendix 1 [file mental-v13-e81213-s001.docx]

# Supplementary Information: Prevalence of cognitive distortion markers in a suicide prevention chat service

In this supplementary information for the manuscript “Prevalence of cognitive distortion markers in a suicide prevention chat service”, we display the variation frequency and prevalence of CDS markers in both the Help seeker and Operator cohorts. Moreover, we present our investigation into using the different types of Help seekers (individuals with suicide ideation vs friends/next of kin) that reach out to the 113 helpline as a control group.

## Monthly CDS counts for the Help seeker cohort

As shown in **Figure S 1** (top left panel), we found a slight increase in both the number of recorded messages and the number of messages that contains a CDS n-gram for the Help seeker cohort. CDS prevalence, which is the percentage of messages that contains a CDS is shown in the top right panel **Figure S 1**, however showed some variation over time, but it is not indicative of systematic growth nor discontinuities or changes relative to the start of the COVID-19 pandemic. In fact, as indicated in the top right panel of **Figure S 1**) CDS prevalence was remarkably stable over timed.

When we stratified the counts and prevalence per CDS category, we found a similar pattern for each category, i.e. a small increase of total number of messages but a stable percentage of all messages over time as is indicated in the lower two panels of **Figure S 1**.


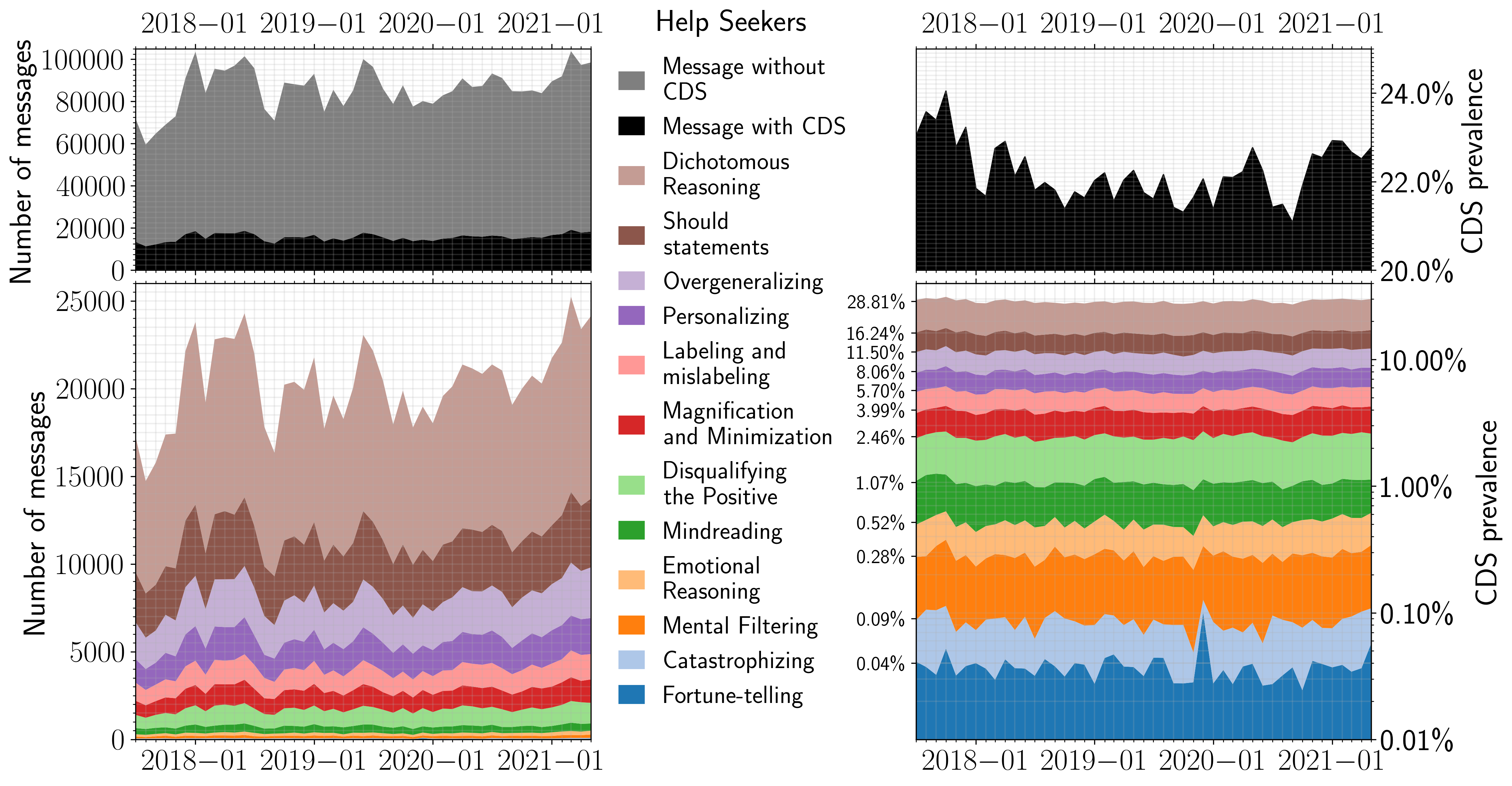


**Figure S 1: CDS prevalence and message volume over time for the Help seeker cohort.** The top left panel shows the monthly number of recorded messages and the top right panel shows the monthly CDS prevalence in the help seeker cohort. The bottom left panel shows the monthly number of recorded messages that contain a CDS from a given category and the bottom right panel shows the monthly CDS prevalence stratified per CDS category in the help seeker cohort. The annotations on the left axis of the bottom right panel indicate the values CDS prevalence per category of the entire cohort. Note that a message could be counted in multiple CDS categories at once.

## Monthly CDS counts for the Operator cohort

As shown in **Figure S 2** (top left panel), we found a slight increase in both the number of recorded messages and the number of messages that contains a CDS n-gram for the Operator cohort. CDS prevalence, which is the percentage of messages that contains a CDS is shown in the top right panel **Figure S 2**, also showed a clear increase during the COVID-19 pandemic. Despite this increased CDS prevalence remained remarkably stable over time across most of the CDS categories.


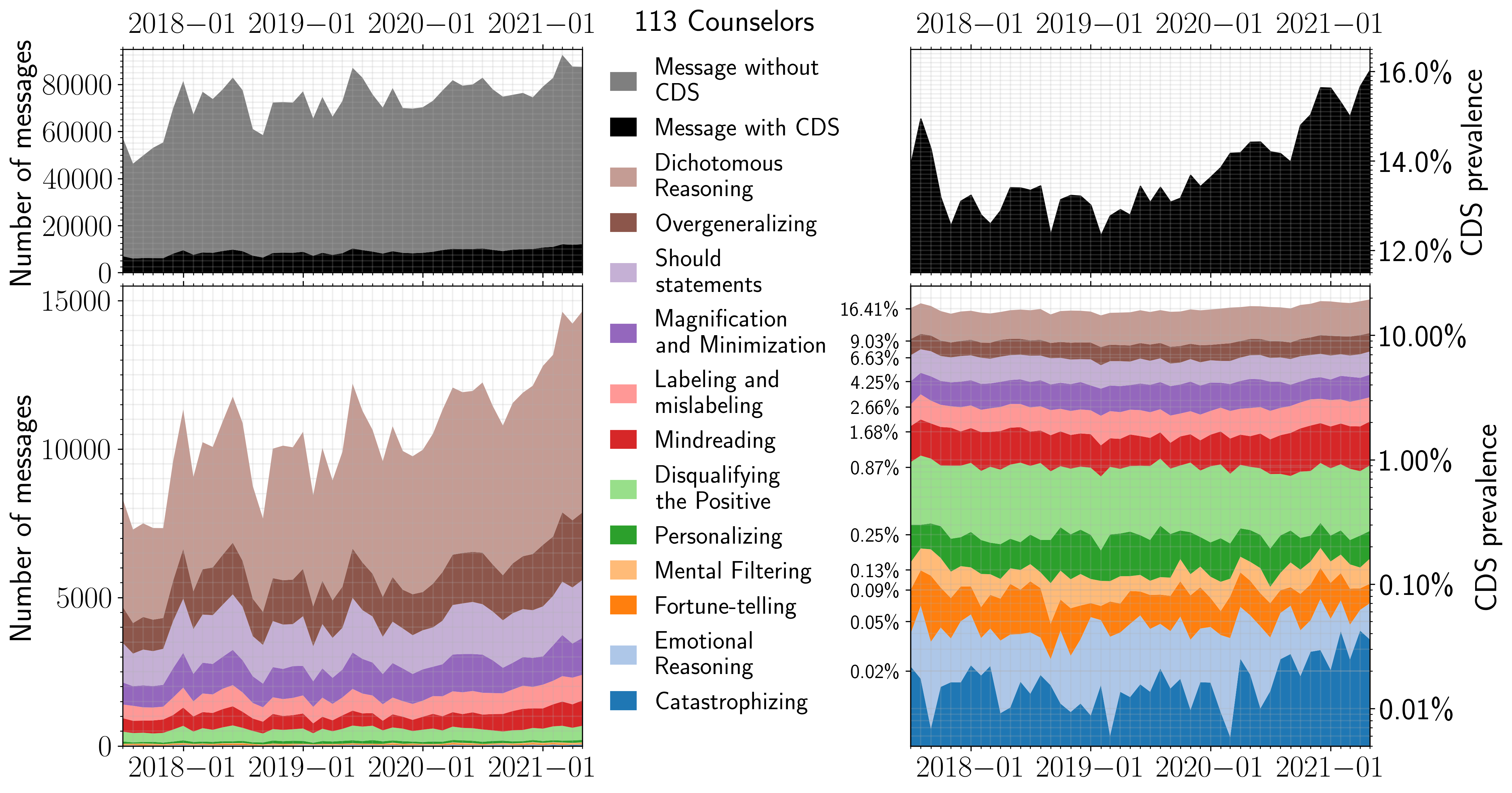


**Figure S 2: CDS prevalence and message volume over time for the Operator cohort.** The top left panel shows the monthly number of recorded messages and the top right panel shows the monthly CDS prevalence in the operator cohort. The bottom left panel shows the monthly number of recorded messages that contain a CDS from a given category and the bottom right panel shows the monthly CDS prevalence stratified per CDS category in the operator cohort. The annotations on the left axis of the bottom right panel indicate the values CDS prevalence per category of the entire cohort. Note that a message could be counted in multiple CDS categories at once.

## Types of Help seekers as control

In addition to individuals with suicide ideation, the counselors at the 113 chat service typically also talk to friends and next of kin of individuals with suicide ideation that want to know more about what they can do. As the database did not have any information logged with regards to whether a help seeker is an individual with suicide ideation or a friend/next of kin. However, we did have the answers that the help seekers provided to the questionnaire, which consists of 9 questions that assess different suicide risk factors [1, 2], that they filled in when they clicked the “chat with us” button on the 113 website. All items in the questionnaire were scored using a double Likert scale. Due to the phrasing of the question that assessed an individuals will to live (i.e., “Ik heb de wil te leven”), the scores of this question were inverted relative to all other inputs. We chose to operationalize our selection between visitors to the chat service between individuals with suicide ideation and friends/next of kin by looking at the total score that they received on the adjusted questionnaire. We assigned a help seeker to the individuals with suicide ideation (denoted by $+$) if they obtained the maximum score ($9\times7=63$) for the questionnaire. Alternatively, we assigned a help seeker to the friend/next of kin cohort (denoted by $-$) if they obtained the minimum score ($9\times1=9$) for the questionnaire. Based on these criteria, we selected $1,722$ individuals for the cohort of individuals with suicide ideation and $528$ individuals for the cohort of individuals of friends/next of kin.


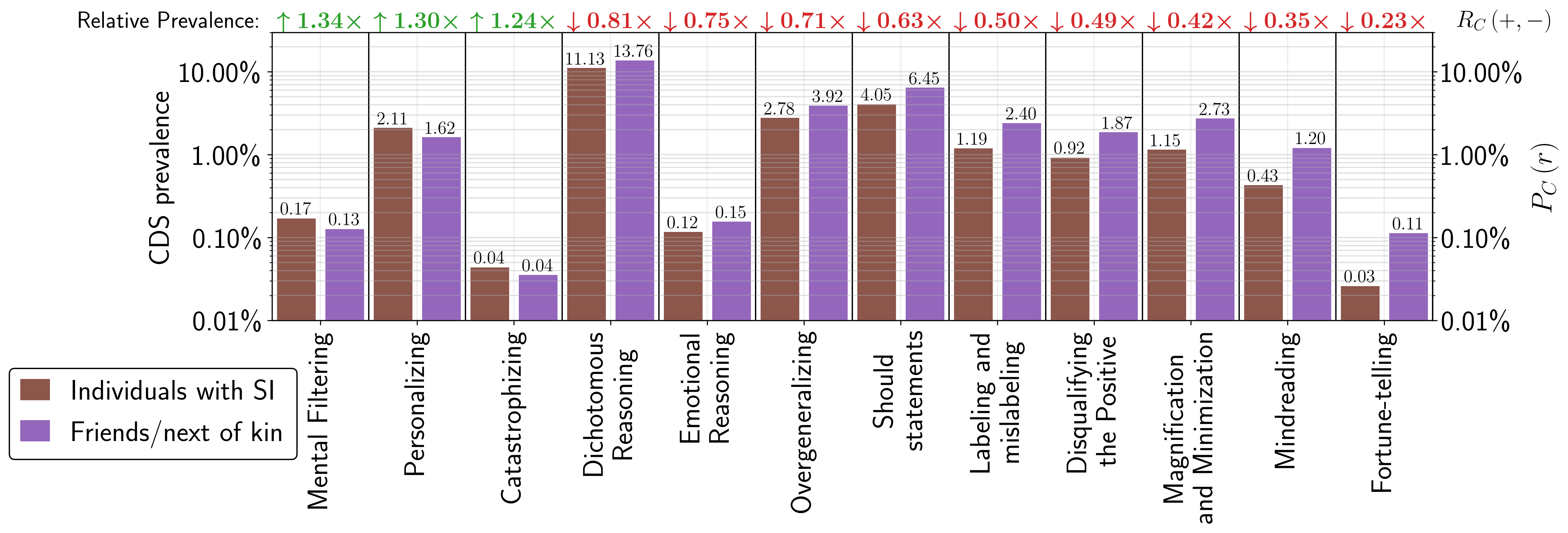


**Figure S 3: CDS prevalence for individuals with suicide ideation (SI) and friends/next of kin stratified per category.** CDs types are ranked left to right by magnitude of CDS prevalence ratio. Each set of 2 bars indicates the CDS prevalence $P_{C}$ for the type $C$, separate by individuals with suicide ideation ($P_{C}\left( + \right)$, brown) and the friends/next of kin cohort ($P_{C}\left( - \right)$, purple) respectively. The prevalence ratio ($R_{C}=\frac{P_{C}\left( + \right)}{P_{C}\left( - \right)}$) for each category $C$ is listed at the top with up- and down-arrows indicating whether the CDS of a particular category is either more or less prevalent in the Help seeker cohort than the Counselor cohort for that category.

**Figure S 3** displays the CDS prevalence calculated across both cohorts. Counter to our initial believe, we saw that only two categories were used three times less by individuals with SI as compared to friends/next of kin, i.e., Mindreading and Fortune-telling. The fact that these two categories differed the most, can be explained by the fact that the latter cohort will be telling the story from their perspective, which forces them to use many of the phrasings that are contained in these two categories. In addition to the prevalence ratio, we also investigated the *within*-session CDS prevalence between the three cohorts.


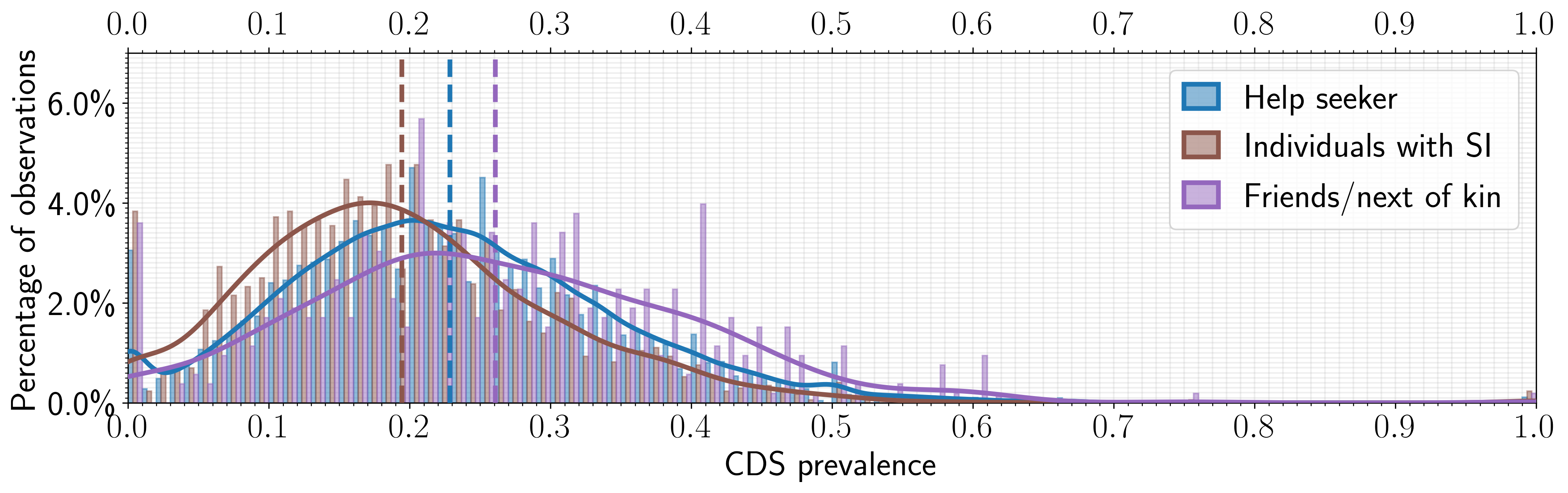


**Figuur S 4: Within-session CDS prevalence distributions for the Help seeker (with** $N=71,148$ **chat-sessions), the Individuals with suicide ideation (SI) (with** $N=1,722$ **chat-sessions), and the Friends/next of kin (with** $N=528$ **chat-sessions) cohorts.** The bars display the distributions of the observations in each cohort. The solid lines display the kernel density estimate (KDE) based on the observations in each cohort. The dashed vertical line indicates the mean CDS prevalence.

**Figure S 4** displays the distribution of the *within*-session CDS prevalence across the three cohorts. Although clear differences in the distributions were visible, we did not find the clear differences with respect to the CDS prevalence that we expected to find. This was likely caused by the fact that chat sessions in the friends/next of kin cohort also describe the same situations as the chat sessions in the individuals with SI cohort. With this in mind, we chose to not use the division between individuals with SI and friends/next of kin as a control in our analysis.

## References

1. Janssen, W., et al., *Can Outcomes of a Chat-Based Suicide Prevention Helpline Be Improved by Training Counselors in Motivational Interviewing? A Non-randomized Controlled Trial.* Frontiers in Digital Health, 2022. **4**.

2. Salmi, S., et al., *The Most Effective Interventions for Classification Model Development to Predict Chat Outcomes Based on the Conversation Content in Online Suicide Prevention Chats: Machine Learning Approach.* JMIR Ment Health, 2024. **11**: p. e57362.
